# Investigating the genetic control of plant development in spring barley under speed breeding conditions

**DOI:** 10.1007/s00122-024-04618-9

**Published:** 2024-04-30

**Authors:** Nicola Rossi, Wayne Powell, Ian J. Mackay, Lee Hickey, Andreas Maurer, Klaus Pillen, Karen Halliday, Rajiv Sharma

**Affiliations:** 1 Scotland’s Rural College (SRUC), Kings Buildings, West Mains Road, Edinburgh, EH9 3JG UK; 2 Institute of Molecular Plant Sciences, School of Biological Sciences, University of Edinburgh, Edinburgh, EH9 3BF UK; 3 Queensland Alliance for Agriculture and Food Innovation, The University of Queensland, Brisbane, Australia; 4 Chair of Plant Breeding, Martin-Luther-University Halle-Wittenberg, Betty-Heimann-Str. 3, 06120 Halle, Germany

## Abstract

**Key message:**

This study found that the genes, PPD-H1 and ELF3, control the acceleration of plant development under speed breeding, with important implications for optimizing the delivery of climate-resilient crops.

**Abstract:**

Speed breeding is a tool to accelerate breeding and research programmes. Despite its success and growing popularity with breeders, the genetic basis of plant development under speed breeding remains unknown. This study explored the developmental advancements of barley genotypes under different photoperiod regimes. A subset of the HEB-25 Nested Association Mapping population was evaluated for days to heading and maturity under two contrasting photoperiod conditions: (1) Speed breeding (SB) consisting of 22 h of light and 2 h of darkness, and (2) normal breeding (NB) consisting of 16 h of light and 8 h of darkness. GWAS revealed that developmental responses under both conditions were largely controlled by two loci: *PPDH-1* and *ELF3*. Allelic variants at these genes determine whether plants display early flowering and maturity under both conditions. At key QTL regions, domesticated alleles were associated with late flowering and maturity in NB and early flowering and maturity in SB, whereas wild alleles were associated with early flowering under both conditions. We hypothesize that this is related to the dark-dependent repression of *PPD-H1* by *ELF3* which might be more prominent in NB conditions. Furthermore, by comparing development under two photoperiod regimes, we derived an estimate of plasticity for the two traits. Interestingly, plasticity in development was largely attributed to allelic variation at ELF3. Our results have important implications for our understanding and optimization of speed breeding protocols particularly for introgression breeding and the design of breeding programmes to support the delivery of climate-resilient crops.

**Supplementary Information:**

The online version contains supplementary material available at 10.1007/s00122-024-04618-9.

## Introduction

The world's food demand is expected to rise significantly by 2050, by as much as 56% (van Dijk et al. 2021). This increase is primarily due to the combined effects of climate change, population growth, and global food supply disruptions. To meet this demand, it is essential to increase crop yields sustainably (Smith 2013). This has been achieved in the past through a combination of improved management practices and the generation of superior germplasm (Cooper et al. 2020; Fradgley et al. 2023). However, recent advances in high-throughput genotyping and phenotyping technologies have opened up new opportunities to accelerate the rate of genetic gain in crops (Li et al. 2018). By reducing crop generation time and using marker-assisted selection and genomic prediction, breeders can now significantly increase the yield potential of crops (Gosal et al. 2020). This has prompted breeders to accelerate the seed to seed time of crops through the deployment of technologies to support rapid generation cycling, such as shuttle breeding, single seed descent, double haploid, and, more recently, speed breeding (Watson et al. 2018a, b).

Prior to speed breeding, the main technology for faster breeding cycles was the use of double haploid technology (DH), which quickly generated homozygous lines after F1 or F2 generations. However, DH has two main drawbacks: Firstly, it requires expensive tissue culture laboratories; secondly, DH populations derive from low recombination events and cross-over rates, which increases the population size needed (Inagaki et al. 1998). Moreover, its effectiveness varies among genotypes as often many genotypes are non-responsive to tissue culture (Hooghvorst and Nogués 2021). Alternatively, single seed descent (SSD) method was adopted in many crops. Traditionally, the SSD approach involves advancing each F2 individual through selfing in a controlled environment with a 16-h photoperiod for long-day plants, achieving up to three generations per year. This method comes with reduced costs and higher genetic variability compared to DH breeding. The increased genetic diversity that results from SSD contributes to improved selection efficiency and serves as a protective measure against genetic drift. As a result, SSD is a powerful tool for enhancing the overall efficacy and success of crop improvement programmes. However, SSD is not always a superior alternative to DH as it leads to slower development of recombinant inbred lines (Caligari et al. 1987; Powell et al. 1986).

Although research on the effects of extended photoperiod on plant growth and development began almost a century ago (Arthur et al. 1930; Garner and Allard 1927), it was not until recently that researchers began to investigate the most efficient combination of environmental factors for reducing the breeding cycle. Watson et al. (2018a, b) demonstrated that speed breeding methods could be adapted to reduce generation time for a broad range of crop species. Developing an efficient speed breeding protocol involves optimizing several environmental factors, with a key one being the exposure to prolonged photoperiods for long-day species. Speed breeding can be integrated with other technologies to achieve different breeding objectives such marker-assisted selection (MAS) for simple traits as genomic selection (GS) for complex traits (Hickey et al. 2017a, b, 2019; Pandey et al. 2022). A body of research has advanced speed breeding protocols aiming to reduce the breeding cycle in long- and short-day plants using controlled environments (Cazzola et al. 2020; Chiurugwi et al. 2019; Fang et al. 2021; Watson et al. 2018a, b; Hickey et al. 2017a, b; Mobini et al. 2020; Samineni et al. 2020; Schilling et al. 2023). Depending on the species, an optimized protocol can reduce the time from crossing to testing to 18 months or 2 years, much shorter than SSD or shuttle breeding. Furthermore, speed breeding offers significant advantages over DH technology as it maintains higher recombination and cross-over events while still achieving a similar reduction in generation time at a reduced cost. As a result, rapid cycling protocols have become popular in plant breeding programmes around the world.

Despite the recent success of speed breeding, there are still opportunities for refinement by optimizing energy and management costs, as the tool is still in its infancy. This is intertwined to the limited understanding underlying the genetics of plant development under such conditions. Specifically, we do not know whether flowering and maturity under very long days (e.g. 22-h light in speed breeding conditions) is genotype dependent and under different genetic controls compared to standard long days (e.g. 16-h days). Understanding this could help breeders and researchers to develop more effective speed breeding protocols. Enhancing our knowledge on this matter can significantly influence the decision-making process for breeders and researchers when considering the adoption of this technology, leading to more effective and targeted crop improvement and research strategies. While studies on speed breeding in cereals have shown that plant development can be accelerated under these conditions (Cha et al. 2022; Watson et al. 2018a, b), experiments have mainly focussed on modern or elite germplasm. As introgression breeding is becoming a valuable tool for gaining access to wild genetic diversity that can help crops adapt to climate change (Gramazio et al. 2021; Hao et al. 2020; Hernandez et al. 2020; Khan et al. 2023; Zhang et al. 2023). Therefore, a better understanding of the genetics of plant development under different long-days conditions would help pre-breeders develop protocols that are effective in speed breeding programmes.

To shed light on the genetic basis of speed breeding, the present study examined the “Halle Wild Barley” (HEB-25) nested associated mapping (NAM) population, which segregates for both wild and domestic alleles (Maurer et al. 2015). The lines were phenotyped for key developmental traits under both speed breeding (22 h of light and 2 h of darkness) and normal breeding (16 h of light and 8 h of darkness). Data from these experiments and whole-genome marker data using the Infinium iSelect 50k SNP chip (Maurer and Pillen 2019) were used in genome-wide association (GWAS) to identify genetic loci associated with the differential responses of spring barley lines grown under the two different artificial growth conditions. To our knowledge, this is the first study to identify the genetic basis of plant development under speed breeding, providing insights into the mechanisms controlling barley’s development-related traits under long and very long days. The results of this study have important implications for the deployment of speed breeding to accelerate the utilization of genetic diversity, particularly wild relatives, to support the development of future crops.

## Material and methods

### Plant material

The present study uses the multiparent nested associated mapping (NAM) population “Halle Wild Barley” (HEB-25), developed by Maurer et al. (2015). This population was generated using 25 wild barley parents (24 *Horderum vulgare ssp. spontaneum*, Hsp and 1 *Hourderum vulgare ssp. agriocrithon*) crossed with spring barley cultivar Barke (*H. vulgare ssp. vulgare*, Hv). The resulting generation was backcrossed with the female parent Barke, following three generations of selfing through single seed descent (BC_1_S_3_). Thereupon, the deriving lines were propagated through the 6th generation of selfing (BC_1_S_3:6_). Further details on the population development are provided in Maurer et al. (2015). This multiparent NAM population has become a crucial genetic resource for investigating various essential traits in barley, including stress tolerance and yield (Büttner et al. 2020; Mehnaz et al. 2021; Saade et al. 2016; Sharma et al. 2018; Wiegmann et al. 2019). In our study, we aimed to efficiently evaluate the HEB-25 to study the genetics of barley’s plants development under different long-days conditions. However, screening the entire population in a glasshouse posed practical limitations. To overcome this issue, we implemented a random sampling approach to select a subset of 190 genotypes from the population, consisting of three to four genotypes from each of the 25 families present in the population. To select a subset of 190 genotypes from the HEB-25, we employed the RAND() function available in Microsoft Excel version 2010 (MS Office). The use of this function allowed to randomly select three to four lines from each of the 25 families, thus minimizing the potential for bias in our selection process. To ensure that we selected an extensively varied subset, we conducted a principal component analysis (PCA) with Rstudio version 4.2.2. This analysis employed the complete panel along with an SNP matrix consisting of 32,955 markers. Subsequently, the PCA plot was produced using the R-package “ggplot2” (Wichham 2016). As depicted in Fig. S1, our subset comprehensively represents the entire population and exhibits considerable diversity. The selected subset was screened in two subsequent experiment rounds (1st from November 2021 to March 2022 and 2nd from July to October 2022). A set of 12 genotypes were included in both rounds of screening for normalization of the experiments that were subsequently used to combine the data across the two screening rounds via a linear mixed model, as outlined in the “statistical analysis” section.

### Speed breeding experiments and phenotyping

In order to fulfil the aim of this study, we gathered phenotypic data on the development of barley plants under different controlled environmental conditions. To achieve this, the experiments were conducted in a glasshouse located at SRUC's Peter-Wilson campus (55°55′17.386″ N−3°10′42.175″ E) manufactured by CambridgeHOK. By measuring developmental traits of the plants under both conditions, we aimed to gain insights into the genetic characteristics of plant development under speed breeding.

The experimental conditions were meticulously chosen to ensure that the phenotypic data collected accurately represented the impact of the photoperiod length used in speed breeding for cereals. The first glasshouse compartment had a photoperiod of 16 h of light and 8 h of darkness (16:8) (hereon called normal breeding: NB), while the second compartment was set up for speed breeding and had a photoperiod of 22 h of light and 2 h of darkness (22:2) (hereon called speed breeding: SB). The temperature in both compartments was programmed at 22 degrees Celsius during the day and 17 degrees Celsius at night, in accordance with the specifications of Watson et al. (2018a, b). The experimental unit was one plant per 0.3 L pot at a density of sowing of 77 plants/m^2^, with five replicates per genotype in a complete randomized block design (RBD). The plants were distributed across the benches in 50 columns and 10 rows of each treatment. The glasshouse is supplied with 400-W high-pressure sodium light fixtures (Sylvania GroLux). The light intensity and the temperature were measured via a quantum sensor (SKP 200—Skye Instruments) and dataloggers (EasyLog USB), respectively.

A set of 100 HEB lines were sown in November 2021 and another set of 96 HEB lines in July 2022. Twelve HEB lines were cultivated under both experiment rounds for normalization, as detailed in the statistical analysis section.

Our study concentrated on two traits that have high heritability and are essential for the successful completion of barley’s life cycle and development: days to heading (as a proxy for flowering time) and days to maturity. We scored the traits by measuring the number of days it took for the plant to reach growth stages BBCH49 (Heading—HEA) and BBCH92 (Maturity—MAT) using the BBCH scale developed by Lancashire et al. (1991) under both NB and SB conditions. Additionally, we measured phenotypic plasticity, which is defined as the changes exhibited by a genotype when grown in different environments (Laitinen et al. 2019). Hence, in our study, plasticity is the quantification of changes in developmental advancement of a genotype across the two controlled environment conditions. Plasticity was calculated for each genotype as the difference between the trait performances under NB and SB. We utilized these derived traits to identify genetic factors that contribute to the plasticity of HEA and MAT (Plasticity.HEA and Plasticity.MAT) across NB and SB. This is a useful measure of adaptation, particularly in the light of the changing global environment characterized by abiotic stresses. Gaining insights into the genetic basis of differential responses observed in different long-days conditions can help us understand how plant development varies under different light conditions. This understanding can be used to develop speed breeding protocols that are tailored to specific genetic backgrounds or germplasm pools (e.g. elite versus wild).

### Statistical analyses

After checking the phenotypic trait values manually for typographical errors, we excluded outliers exceeding 3 standard deviations in each genotype. Subsequently, we removed genotypes with less than 3 replicates, per environment, from the analysis. Next, we fitted the best linear mixed model to obtain the best linear unbiased estimator (BLUE) for the studied traits, considering genotypes as fixed effects and the experiment round, along with the row and column effects (due to the varying light distribution across benches), as random effect. Cultivating 12 common genotypes across experiment rounds and incorporating this factor into the model enabled the normalization of phenotypic data from both rounds of the experiment. The models were fitted using “lmer” function from the package “lme4” (Bates et al. 2015) in Rstudio version 4.2.2. We then compared different models that considered either row and/or column effects or none of them and selected the best performing model based on the lower AIC (Akaike Information Criterion). The model comparison was made via the “aic” function in the basic package “stats” in Rstudio version 4.2.2.

Summing the genotypes effects to the intercept provided unique values for each genotype which were then used for the GWAS and for calculating plasticity.

Traits’ heritability was calculated using Piepho’s (Piepho and Möhring 2007) method using the R-scripts provided in Covarrubias-Pazaran (2019).

The GWAS was performed using barley 50K SNP markers (Bayer et al. 2017; Maurer and Pillen 2019) by fitting the following model:

$$ {\text{Y}}\, = \,{\text{Xb}}\, + \,{\text{Wm}}\, + \,{\text{Zu}}\, + \,{\text{e}} $$

where** y** is a N × 1 column vector of the BLUE values of phenotypic data of N NAM lines (N = 190 max in our case); b is a vector of population structure effects as fixed effects; X is an incidence matrix relating b to y, consisting of principal components loadings from the PCA; **m** is a vector of fixed marker effects; **W** is a marker matrix containing marker types (as − 1, 0, and 1);** u** is a vector of random polygenic effects where $u \sim MVN\left( {0,{\mathbf{K}}\sigma_{u}^{2} } \right)$, **K** is the additive relationship matrix obtained from the markers using the function “A.mat” in the “rrBLUP” package (Endelman 2011) in Rstudio version 4.2.2: **Z** is an incidence matrix linking** u** to **y**; and **e** is a vector of random residuals where $e \sim MVN\left( {0,{\mathbf{I}}\sigma_{e}^{2} } \right)$ and** I** is the identity matrix.

The correction for population structure was conducted via the kinship correction and using the top 6 principal components as covariates, namely the Q + K model (Isidro-Sánchez et al. 2017). The number of principal components used in the analysis was established from a scree plot and by visually evaluating the component number at which the rate of eigenvalue decrease began to plateau.

GWAS was conducted using SNP with MAF > 0.05, and the threshold of false discover rate (type I error rate) was set at α = 0.05 for each trait. More details on the markers 50k Illumina Infinium iSelect SNP array given in (Maurer and Pillen 2019).

In addition, markers effect size was computed using the “mixed.solve” function in “rrBLUP” using Rstudio version version 4.2.2. The effects are derived from the wild parents’ of the population.

### Analysis of alleles associated with *PPD-H1* and *ELF3*

Genotype groups were created from polymorphisms present at some of the significantly associated markers in the two major QTLs (co-located with the candidate genes *ELF3* and *PPD-H1*) found in the GWAS scans. This yielded four different groups based on the allelic combinations for the SNPs in the two loci. Barke is the only domesticated parent for the HEB-25 and used as reference genome for the SNP computation. Alleles presenting polymorphism to this genome are referred as “Hsp” (from *H. spontaneum*, wild parent) and the Barke ones as “Hv” (from *H. vulgare*, domesticated parent) as shown in Table 1. These four groups were then displayed via boxplot for all the traits. A pairwise Student's t-test was used for detecting differences among the genotypic groups. All the comparisons between the groups were made both in the form of parametric t-test and permuted t-test. The boxplots were created using the package “ggplot2” (Wickham 2016) and t-tests via the “t.test” function in Rstudio version 4.2.2.

**Table 1:** Genotypes groups based on the combinatorial allelic combination from the haplotypic analysis

Group name	Number of genotypes/condition	ELF3 allele	PPD-H1 allele
PPD-H1Hv|ELF3Hv	125	Domesticated (Hv)	Domesticated (Hv)
PPD-H1Hv|ELF3Hsp	28	Wild (Hsp)	Domesticated (Hv)
PPD-H1Hsp|ELF3Hv	17	Domesticated (Hv)	Wild (Hsp)
*PPD-H1Hsp|ELF3Hsp*	*3*	*Wild (Hsp)*	*Wild (Hsp)*

Hv: domesticated allele and Hsp: wild allele. These haplotypes are derived from SNPs present in the original haplotypes harboured in the HEB25 families

## Results

### Plant development acceleration due to speed breeding is genotype dependent

In general, plants completed their life cycles faster in SB than in NB. Flowering (HEA) occurred 36 ± 7 days after germination in SB conditions and 52 ± 11.5 days in NB conditions. This corresponds to a 15.9 ± 6.88-day developmental acceleration under SB. However, the average difference in days to maturity (MAT) between the two conditions was 7.7 ± 6.88 days. Figure S2 shows the distribution of these traits as frequency histograms. Plasticity.MAT exhibits an uncommon trait distribution due to the convergence of numerous genotypes at maturity levels in both MAT SB and MAT NB, as depicted in Fig. S2.

BLUE values for HEA and MAT and their derived plasticity traits, along with the summary statistics for mean, standard deviation, minimum and maximum values, and heritability values, are provided in Data S1 and Table S1, respectively.

Notably, a significant proportion of the lines (approximately 90%) flowered and matured earlier under SB than under NB. This suggests that there is substantial variation in trait values and that SB has an important effect on plant growth. The substantial amount of genetic variability was observed in how plants responded to both conditions, enabling a GWAS to be conducted as described in the subsequent section. The heritability values of these traits were high, albeit lower in SB than in NB (Table S1).

### The effect of allele combinations associated with *ELF3 *and *PPD-H1* on MAT, HEA, and plasticity

**Fig. 1:**
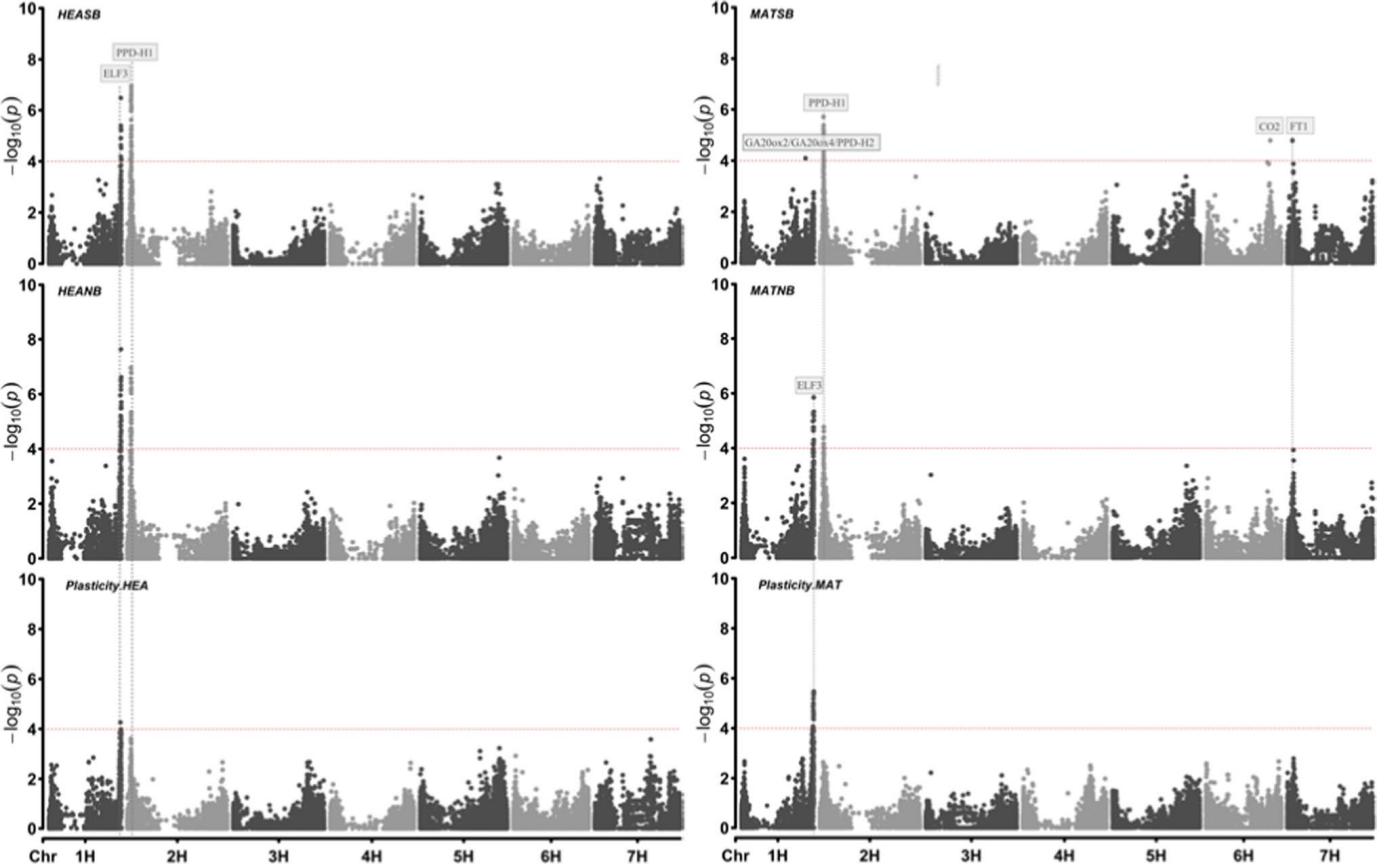
Manhattan plots from the three traits (heading (HEA), maturity (MAT), and plasticity in two photoperiod conditions (normal breeding and speed breeding)). Seven barley chromosomes are shown (1H-7H) horizontally, and –log10(p-values) are displayed vertically on the y-axis. Significant FDR threshold grey dashed line set at 0.05 (− log10 p-value). Flowering-time candidate genes are shown in the rectangle boxes. Plots were created using the “CMplot” package (Yin et al. 2021) in Rstudio version 4.2.2. The details of the significant peaks and the markers underlying these peaks are provided in Data S2

To better understand the genetic factors that control plant development under SB and NB conditions, a GWAS was conducted for each trait. We focussed on identifying QTLs associated with the regulation of the development under SB and NB conditions. Specifically, six GWAS scans were performed across four primary traits: HEA in NB, HEA in SB, MAT in NB, and MAT in SB and their corresponding plasticity traits: Plasticity.HEA and Plasticity.MAT, across the two conditions. Manhattan plots and the list of markers, their position, the level of association − log_10_(*P-*value) ≥ 4, and their effects are provided in Fig. 1 and Data S2, respectively.

The GWAS scans of HEA and MAT traits under both SB and NB revealed two prominent QTLs that are co-located with the major flowering-time genes *ELF3* on chromosome 1H and *PPD-H1* on chromosome 2H (Russell et al. 2016), implicating their central importance in the control of flowering and maturity. Interestingly, the *PPD-H1* association with MAT was maintained under both NB and SB. Conversely, for the plasticity traits, only the *ELF3* association remained significant, highlighting its involvement in governing the plasticity of HEA and MAT under SB conditions. In a previous study (Parrado et al. 2023), it was noted that PPD-H1 was associated with plasticity of flowering time under different photoperiod regimes. In the current study, the association of PPD-H1 with Plasticity.HEA was just below the significance threshold.

Our GWAS results emphasize the importance of major flowering-time genes in barley for the regulation of HEA and MAT traits, which aligns with the previous studies (He et al. 2019; Maurer et al. 2015, 2016). However, our findings also highlight the wider relevance of these two genes specifically in the context of speed breeding, which has not been previously reported in the literature. Moreover, we have identified three more genomic regions (1H, 6H, and 7H) for MAT in speed breeding. On chromosome 1H, this region may correspond to the candidate genes PPD-H2, GA20ox2, and GA20ox4. The peak on chromosome 6H is near the candidate gene CONSTANS 2, and on chromosome 7H, it is close to FLOWERING LOCUS T. The detection of these QTLs in SB is particularly interesting, considering the strong correlation observed between MAT and HEA traits (Fig. S3 and Table S2). The presence of few additional regions of relevance suggests that these specific regions may have a greater influence on the MAT trait under SB, as they do not exhibit significant association in NB. This finding implies the existence of unique genetic mechanisms that regulate MAT trait responses in the context of speed breeding.

The validity of our findings was further supported through the incorporation of major QTL peaks from chromosome 1H and 2H as covariates in our GWAS model (Figure S4 and Data S5). As expected, these two major QTLs disappeared after incorporation as covariates. Consequently, we successfully detected prominent QTL peaks in regions proximal to known flowering-time genes such as FT1 (MAT SB and NB), CO2 (MAT SB), GA20ox2 and *GA20ox4* and PPD-H2 (MAT SB), and PHYC (HEA NB, Plasticity.HEA).

The conspicuous association detected near the *FT1* genomic region on chromosome 7H strongly suggests its importance in regulating MAT in addition to *PPD-H1* and *ELF3*. Furthermore, the significant association of markers in regions other than the ones containing the two main QTLs identified, implies the involvement of the additional genes *PPD-H2*, *GA20ox, PHYC*, and *CO2* in the regulation of these traits.

Overall, our GWAS results shed new light on the role of allelic variation at major flowering-time genes in the control of heading and maturity in barley under speed breeding conditions. Additionally, they reveal the broader significance of these genes in the specific context of speed breeding, providing valuable insights not previously reported in the literature. The identification of QTLs associated with the MAT trait in SB further suggests the involvement of additional genes, highlighting the complexity of this trait and its response to different growth conditions.

### Domesticated alleles at *ELF3 *and *PPD-H1* confer higher plasticity

**Fig. 2:**
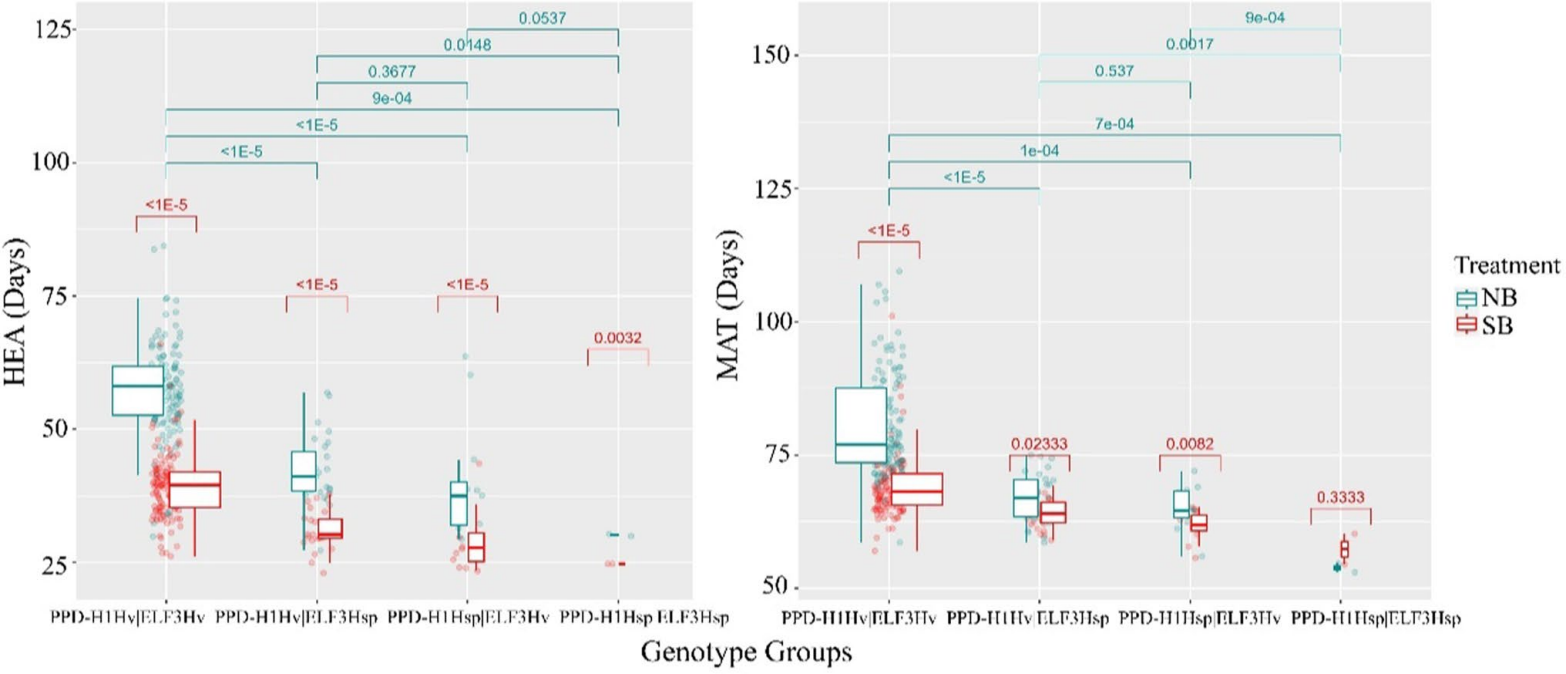
PPD-H1 and ELF3 alleles-based boxplots from HEA **a** and MAT **b**. Boxplots of the response of different genotype groups to MAT and HEA, arising from the combinatorial allelic analysis displayed on the X-axis (PPD-H1_Hv_/ELF3_Hv_, PPD-H1_Hv_/ELF3_Hsp_, PPD-H1_Hsp_/ELF3_Hv_, and PPD-H1_Hsp_/ELF3_Hsp)_. The P-values shown above each allelic class are derived from permutation t-tests as shown in Data S3. For both traits (flowering and maturity), the presence of domesticated alleles tends to delay flowering and maturity under both speed breeding and normal breeding conditions

The combination of alleles at the *ELF3* and *PPD-H1* genes is important under both SB and NB conditions. Different allelic combinations at *ELF3* and *PPD-H1* loci (Table 1, *PPD-H1Hv/ELF3Hv*, *PPD-H1Hv/ELF3Hsp*, *PPD-H1Hsp/ELF3Hv, and PPD-H1Hsp/ELF3Hsp*) also affect MAT, HEA, and their plasticity. As observed by Maurer et al. (2015) and Zahn et al. (2023), genotypes carrying at least one wild allele at one of the two loci tend to flower earlier compared with lines carrying domesticated alleles under both loci (*p* values <  = 0.0009, Fig. 2). This is consistent with the wild alleles effect of the significant markers found in our GWAS scan, as their effect is negative on the traits value, accelerating the plant’s development (Data S2).

**Fig. 3:**
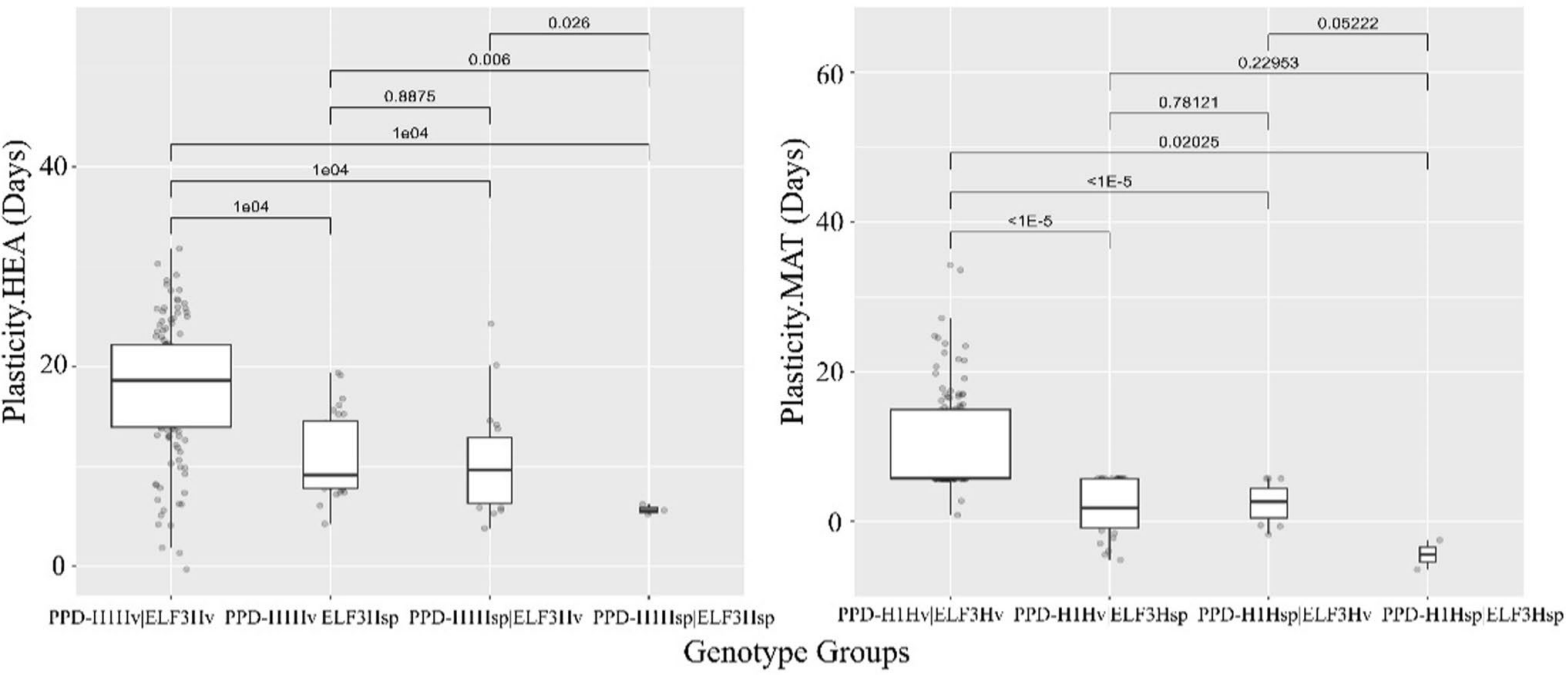
PPD-H1 and ELF3 alleles-based boxplots from Plasticity.HEA **a** and Plasticity.MAT **b**. Boxplots of the response of different genotype groups to Plasticity.HEA and Plasticity.MAT, arising from the combinatorial allelic analysis. PPD-H1_Hv_/ELF3_Hv_, PPD-H1_Hv_/ELF3_Hsp_, PPD-H1_Hsp_/ELF3_Hv_, and PPD-H1_Hsp_/ELF3_Hsp_ are shown on the X-axis. The significance values shown are derived from permutation t-tests as shown in Data S3. The presence of wild alleles at PPD-H1 and ELF3 tends to reduce the levels of plasticity for flowering and maturity times across both speed breeding and normal conditions

Domesticated haplotypes at both loci appear to give higher levels of plasticity compared to the wild haplotypes (*p* values <  = 0.02, Fig. 3 and Data S3a). Nevertheless, the means between experimental conditions within all genotypes’ groups are significantly different except for genotype group 4’s MAT. Therefore, SB reduced the HEA and MAT in all the genotypes studied; however, looking at the means in Data S3b and Fig. 3, the extent of cycling acceleration in genotypes carrying wild alleles is very low compared to the ones harbouring domesticated alleles, especially for MAT. Hence, wild alleles at *PPD-H1* and *ELF3* confer early flowering under both conditions.

To test whether breeders would unintentionally select for specific alleles at PPD-H1 and ELF3 when excluding late maturing plants under speed breeding, we ranked genotypes based on their MAT values under SB and classified the late maturing genotypes into wild or domesticated allelic classes. Later maturing plants in the 75th percentile harboured domesticated alleles at both the *ELF3* and *PPD-H1* loci (Data S5 and Fig. S5), suggesting that SB could result in the distortion of allele frequencies at these two loci during generation advance.

## Discussion

To our knowledge, this is the first time that the genetic control of speed breeding has been explored. Various studies have described the benefits of speed breeding (Ahmar et al. 2020; Bhatta et al. 2021; Bohra et al. 2020; Pandey et al. 2022; Samantara et al. 2022; Song et al. 2022; Wanga et al. 2021) and optimized protocols for the deployment of speed breeding (Cazzola et al. 2020; Chiurugwi et al. 2019; Fang et al. 2021; Watson et al. 2018a, b; Hickey et al. 2017a, b; Mobini et al. 2020; Samineni et al. 2020; Schilling et al. 2023) but the genetic basis of plant development under such conditions remains unexplored. In this study, we adopted a GWAS approach to unravel the genetic control of speed breeding in a barley NAM population grown under two growth conditions, one with a photoperiod of 22 h of light 2 h of darkness (SB) and the other on 16 h of light and 8 h of darkness (NB). By studying a subset of the spring barley HEB 25 NAM population (Maurer et al. 2015), a broad range of wild and domesticated alleles were explored and led to the identification of candidate genes associated with the control of developmental traits under both SB and NB. Two significant candidate genes were pinpointed: *ELF3* and *PPD-H1* controlling both days to heading and days to maturity. Most importantly, by measuring the changes exhibited by an individual genotype over the two treatments, we were able to derive an estimate for plasticity and ascribe a candidate gene *ELF3* that is strongly associated with it, supporting its role as a key hub integrating gene networks influencing overall plasticity (Laitinen et al. 2019).

The previous studies have shown that the *PPD-H1* and *ELF3* genes are involved in the genetic control of several agronomic traits in barley (Digel et al. 2016; Ejaz and von Korff 2017; Gol et al. 2021; Ochagavía et al. 2022). At the *PPD-H1* locus, a variety of natural variants have been identified and categorized into two distinct groups: the sensitive allele, *Ppd-H1* which reduces flowering time during long days and the insensitive variant, *ppd-H1* which delays flowering in long days (Russell et al. 2016; Turner et al. 2005 Fernández-Calleja et al., 2021). The former variant likely represents the ancestral allele, found in HEB-25, and present in winter and Australian barleys (Hu et al. 2023) whereas the *ppd-H1* allele is prevalent in Barke and numerous European and North American spring barley cultivars. With respect to *ELF3*, knowledge and understanding of the phenotypic effects of the allelic series is less established than for *PPD-H1*. Faure et al. (2012) were the first to discover a loss of function allele at this locus, and they identified *ELF3* as the candidate gene responsible for the *eam8* mutant originating from the Scandinavian-induced mutation experiments performed in the past century (Lundqvist 2009). This allele confers early flowering both in short and long days, compared to the domesticated allele. Such a response is similar to the one observed in our study and is consistent with the findings from Zahn et al. (2023) and Zhu et al. (2023).

Both *PPD-H1* and *ELF3* are linked to the expression of *FT1* and *GA20ox* genes that are downstream floral integrators that control the flowering response (Boden et al. 2014; Campoli et al. 2012; Cheng et al. 2023; Faure et al. 2012; Turner et al. 2005). *ELF3* delays the flowering response whereas *PPD-H1* accelerates the response in long days. In Arabidopsis, *ELF3* is a repressor of *PRR7* (Dixon et al. 2011; Herrero et al. 2012; Nusinow et al. 2011), which is an homologue of *PPD-H1*. In barley, this interaction has been studied at the transcript level (Faure et al. 2012; Zahn et al. 2023) with both the alleles present in *eam8* mutant and the wild *ELF3hsp* correlate with a higher *PPD-H1* expression that leads to early flowering compared to the domesticated *ELF3hv* variant. Furthermore, Müller et al. (2020) hypothesized that, as seen in Arabidopsis, *ELF3* antagonizes the light input in the circadian clock during the night. Such response would explain why the domesticated *ELF3hv* alleles, under NB, confer late flowering, however, under SB such genotypes accelerate plant development more significantly compared to genotypes that harbour wild alleles, as in the latter conditions, the night is very short. However, such response it is visible only in a *ppd-H1* background as *Ppd-H1* confers early flowering under both conditions. In this context, the sensitive allele *Ppd-H1* under long days seems to be less influenced by *ELF3*’s suppression. In addition, an independent pathway has been hypothesized where the allele behind *eam8* and the wild *ELF3hsp* allele induces early flowering independently from *Ppd-H1* (Boden et al. 2014; Faure et al. 2012; Zahn et al. 2023). This would explain why we observe early flowering phenotypes in the presence of *ELF3hsp* in a *ppd-H1* background.

In summary, this study has highlighted the importance of both *PPD-H1* and *ELF3* in the control of speed breeding in barley. The deployment of the HEB-25 population enabled alleles at these two loci to be fully explored in the context of speed breeding. Our findings will be particularly important for the deployment of SB in crop improvement programmes that focus on the incorporation of new sources of genetic variation from wild relatives (Gramazio et al. 2021; Hao et al. 2020; Hernandez et al. 2020; Khan et al. 2023; Zhang et al. 2023). Data from this study predict that the deployment of this technology to accelerate generation time in breeding will select against specific alleles in the genomic regions on chromosomes 1 and 2 where *PPD-H1* and *ELF3* are located, resulting in late flowering genotypes not advanced to the next generation. Furthermore, a comparison of the allelic series at *PPD-H1* and *ELF3* (Fig. 3) identified that domesticated alleles at these two loci, which are those that tend to be unintentionally selected against under rapid cycling conditions, also are likely to be associated with higher levels of plasticity. Further work is needed to test these observations under field conditions so that we can identify genotypes that can better withstand a range of climatic conditions (Cockram et al. 2007) and create climate-resilient cultivars. In addition, this study demonstrated that genotypes that harbour wild alleles at *PPD-H1* and *ELF3* exhibit early flowering under both speed breeding and normal conditions. Such knowledge may assist breeders in optimizing the allocation of resources to advance breeding material under speed breeding conditions.

### Supplementary Information

Below is the link to the electronic supplementary material.

Supplementary file1 (XLSX 52 kb)

Supplementary file2 (DOCX 1070 kb)
