# Supplementary material for: Investigating the genetic control of plant development in spring barley under speed breeding conditions
Source: Theor Appl Genet. 2024 Apr 30;137(5):115. doi: 10.1007/s00122-024-04618-9 (PMC11063105; doi:10.1007/s00122-024-04618-9)
Supplement: Supplementary file 2 — Supplementary file2 (DOCX 1070 kb) [file 122_2024_4618_MOESM2_ESM.docx]

**Supplementary Figures**

**
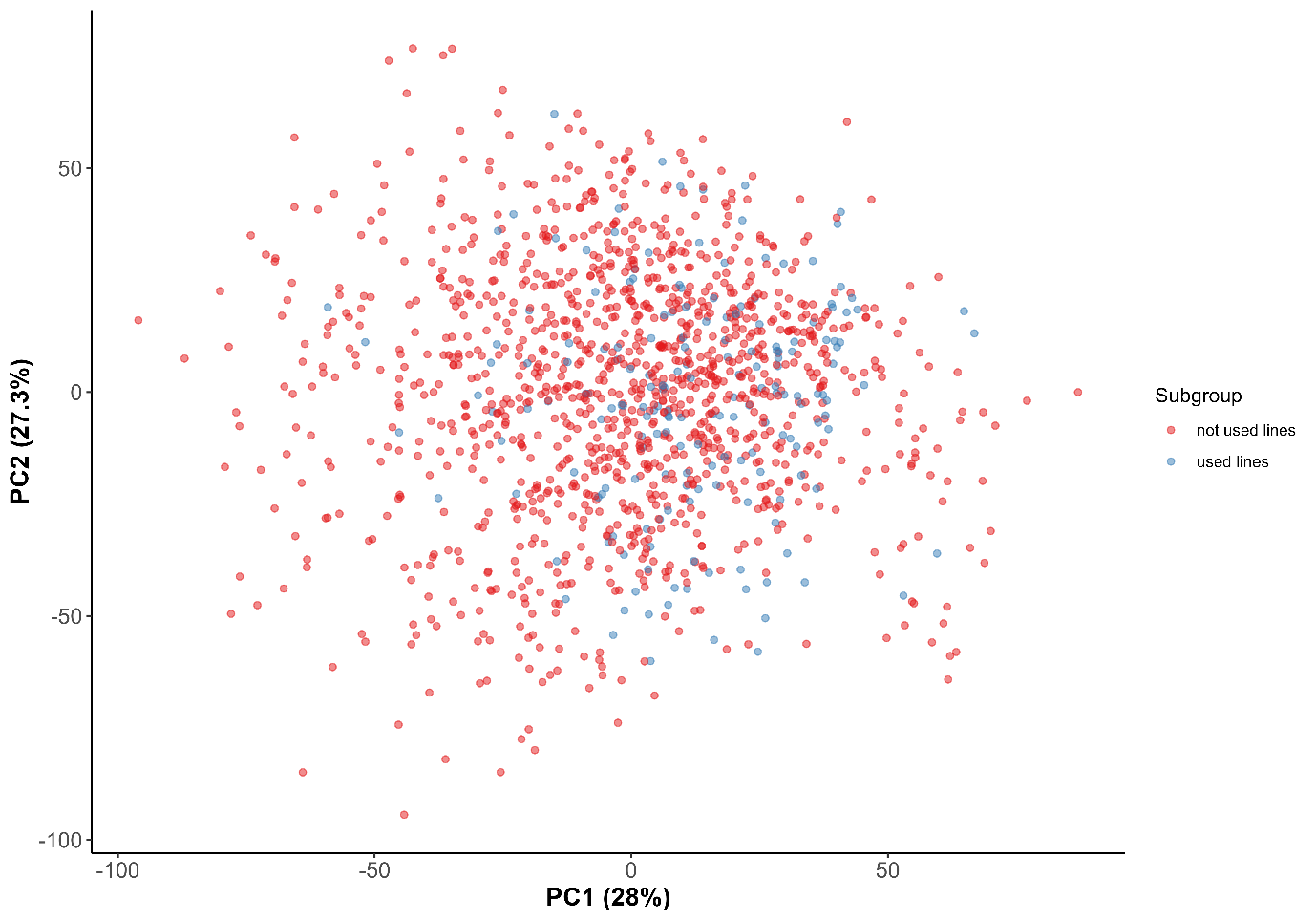
**

**Figure S1**. Principal component analysis for HEB-25 based on 32,955 SNP data. Two principal components (PCs) capturing the largest amount of genetic variation are shown as x- and y-axiss, respectively. Percentages in brackets denote the variance explained by the respective PC. In red are the lines studies and in blue the entire HEB-25 population. The genotypes used for this population (blue) are widely spread across the whole population, confirming the subset used for this study is representive of the HEB-25 population.


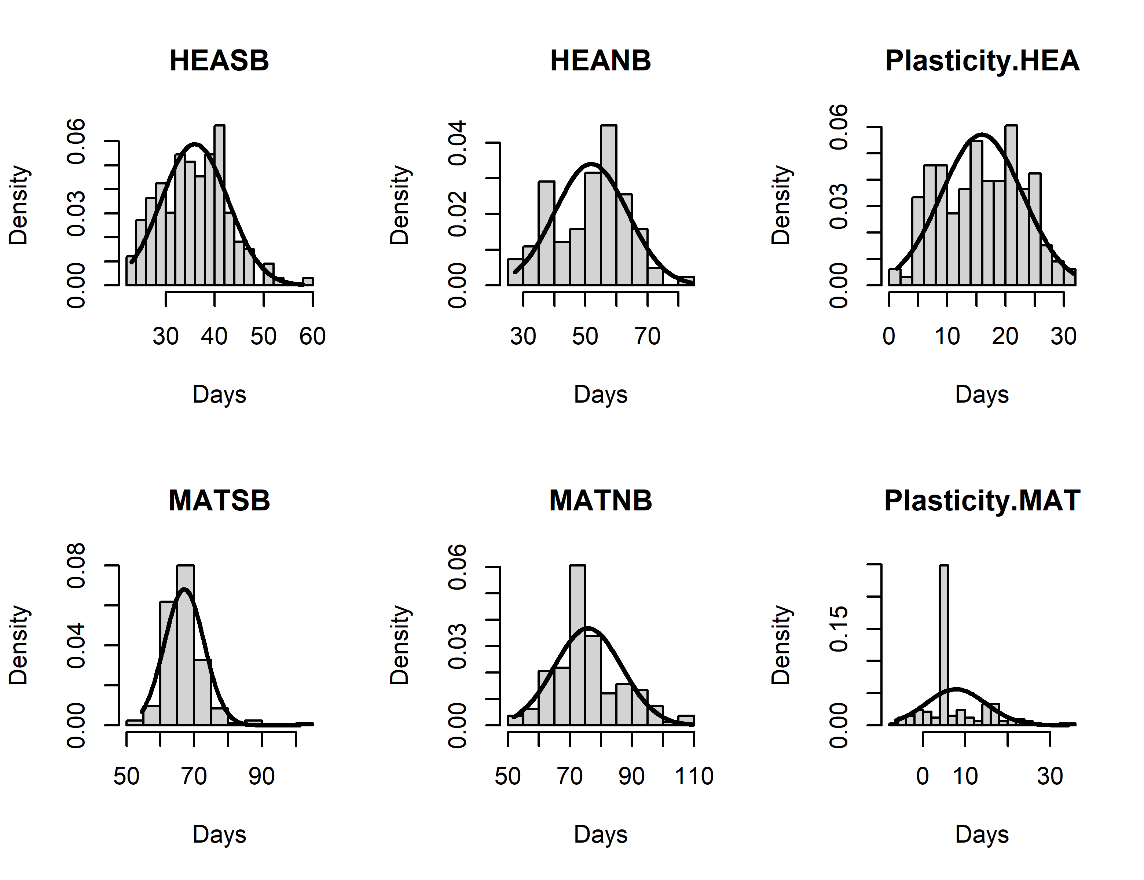


(b)

(d)

(e)

(f)

(c)

(a)

**Figure S2**. Figure showing the frequency distribution of the 6 traits (a-f). The density plots illustrate days on the X-axis and the number of lines in density on the Y-axis. Flowering time and maturity in speed breeding are represented by HEASB and MATSB, while in normal breeding, HEANB and MATNB are used. Plasticity of flowering time and maturity is depicted with abbreviations Plasticity.HEA and Plasticity.MAT, respectively.


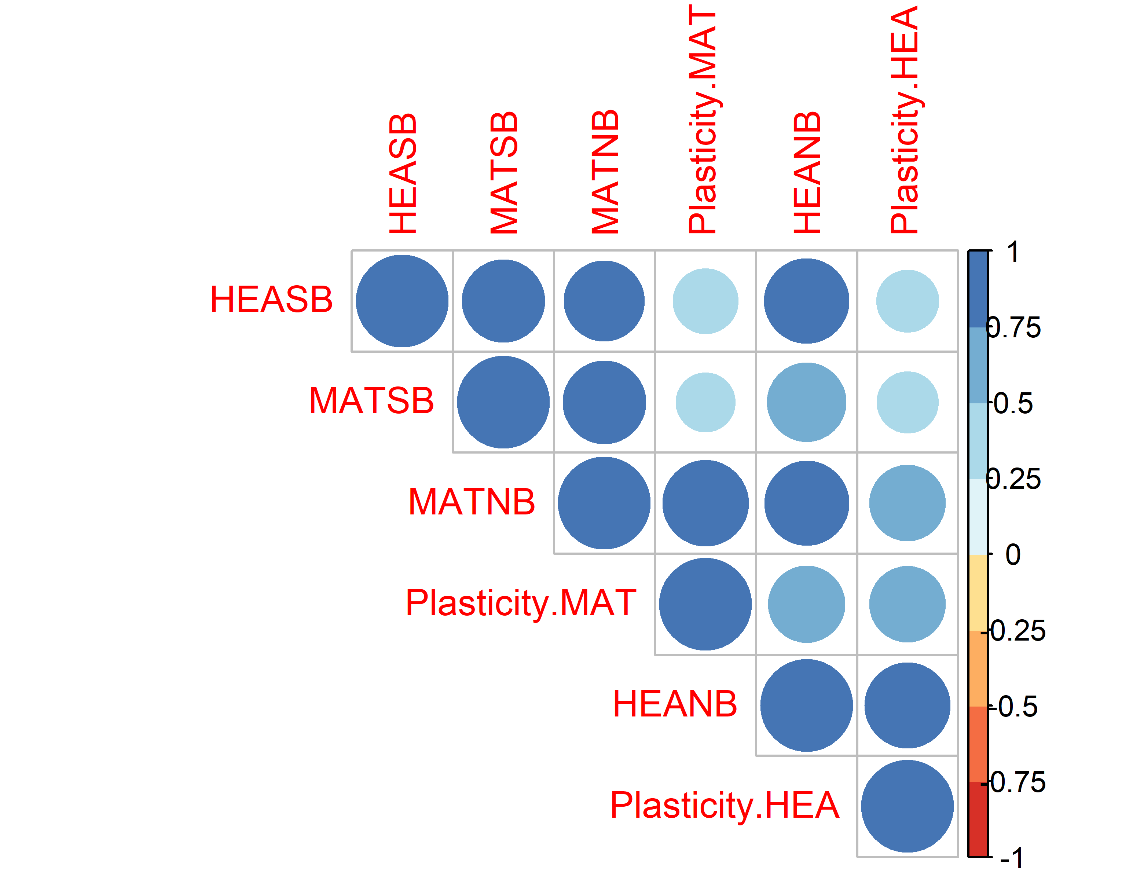


**Figure S3**. The plot displays a correlation matrix (from Table S2), with the six different traits represented along both the x-axis and the y-axis. Flowering time and maturity in speed breeding are represented by HEASB and MATSB, while in normal breeding, HEANB and MATNB are used. Plasticity of flowering time and maturity is depicted with abbreviations Plasticity.HEA and Plasticity.MAT, respectively.Each cell in the matrix corresponds to the correlation coefficient between a pair of traits. The color of each cell represents the strength and direction of the correlation between the two traits. The color scale ranges from blue indicating a negative correlation, through white for no correlation, to a red indicating a positive correlation. The intensity of the color corresponds to the magnitude of the correlation coefficient, with darker shades indicating stronger correlations. The plot was created using the R -package “corrplot” (Wei T, Simko V 2021) in Rstudio version 4.2.2.


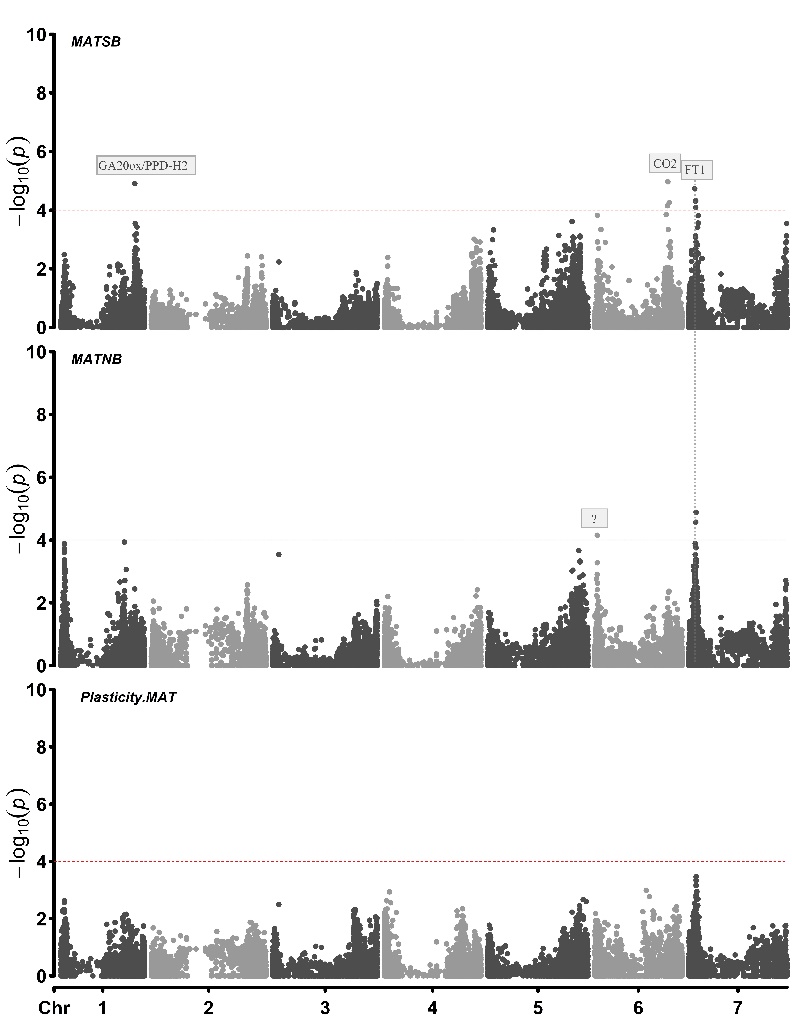

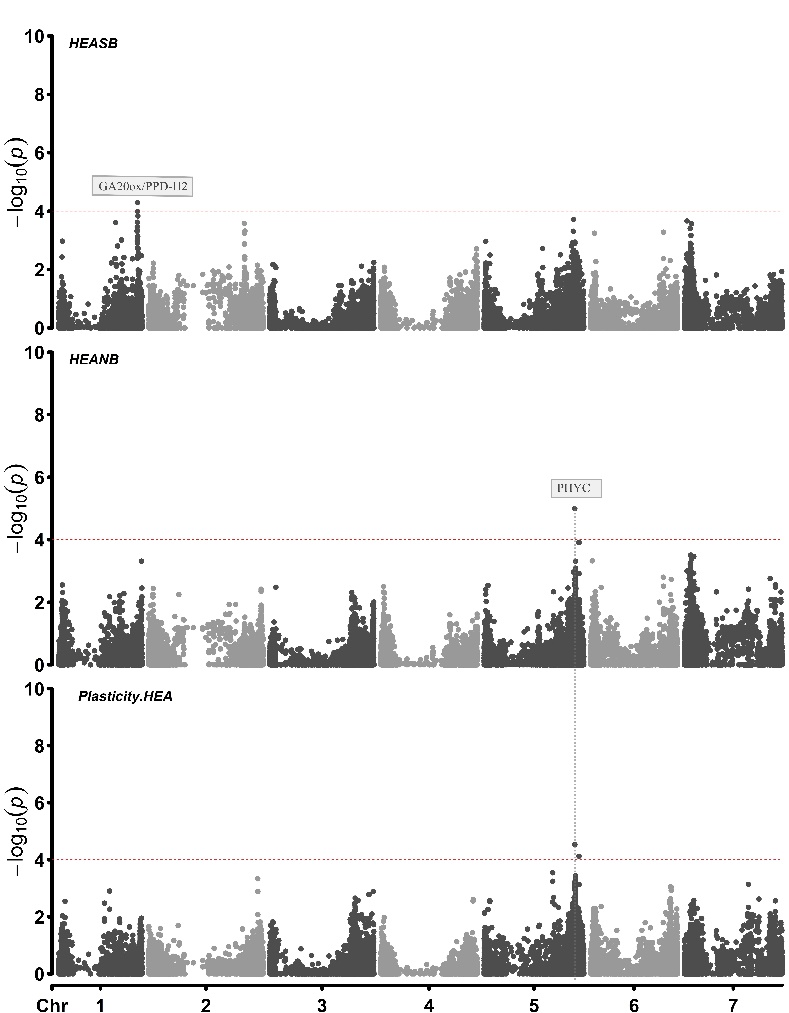


**Figure S4**. Manhattan plots from the six-traits using PPD-H1 and ELF3 as covariates. Seven barley chromosomes are shown (1H-7H) horizontally and –log10(p-values) are displayed vertically by dotted line. Significant threshold dashed line set at –log10 (p-value) = 4.0. The coinciding flowering time candidate genes are shown in the rectangle boxes. Plots were created using the “CMplot” package (Yin et al., 2021) in R studio version 4.2.2. The details of the significant peaks and the markers underlying these peaks are provided in **Supplementary Table S5**.


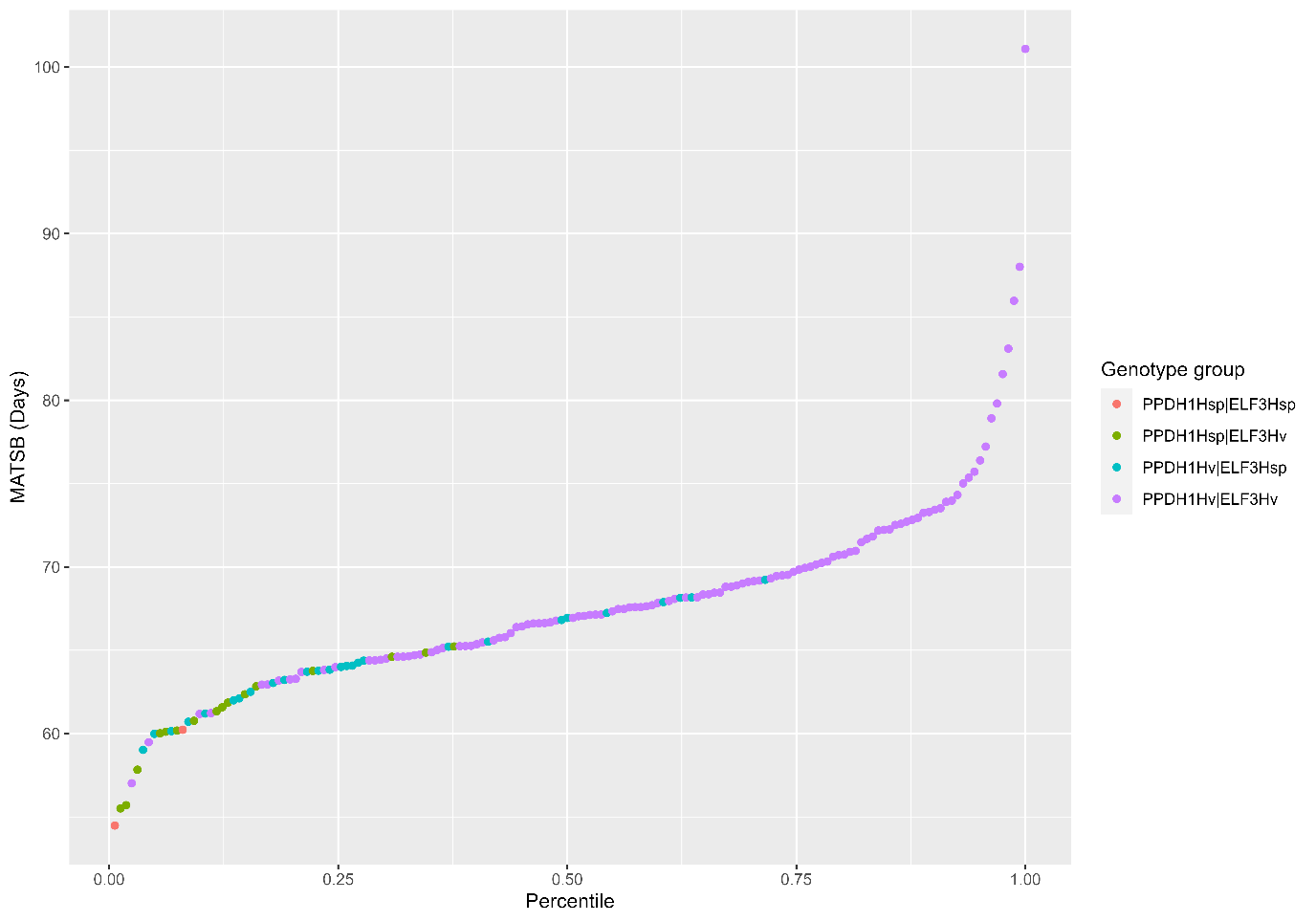


**Figure S5**. The graph displays a distribution of a MAT SB across 162 entries, categorized into four distinct genotype groups. The x-axis represents percentiles. Each point is color-coded to represent one of the four genotype groups, allowing for a visual comparison of their distribution in the dataset.
